# Pet dogs supporting caregivers of children with special needs

**DOI:** 10.6026/973206300220504

**Published:** 2026-01-31

**Authors:** Sandeep Aggarwal, Vaishali Sharma, Jaspreet Singh Virdi, Kanwar Shabaz Singh Sandhu, Chetna Jangwal, Ravleen Kaur

**Affiliations:** 1 Department of Pediatrics, Government Medical College, Amritsar, Punjab, India

**Keywords:** Anxiety, caregiver support, emotional benefits, intellectual disabilities, pet therapy

## Abstract

Caregivers of intellectually disabled children often experience high levels of anxiety and this study examines the potential emotional benefits of pet dog ownership in reducing anxiety among these caregivers. Therefore, it is of interest to examine the impact of pet dog ownership on the emotional well-being of caregivers of intellectually disabled children. Conducted in Amritsar, India, with 112 participants, it divided caregivers into two groups: those who owned a dog and those who did not. Anxiety levels were measured using the Hamilton Anxiety Scale before and after 3-6 months of pet ownership. Results showed a significant reduction in anxiety among dog-owning caregivers compared to non-dog owners. Thus, we show the significant reduction in anxiety levels among caregivers who own dogs, offering new insights into how pet ownership can positively affect caregivers' emotional well-being.

## Background:

Caring for children with intellectual disabilities presents unique challenges that can significantly impact the caregiver's physical and emotional well-being. The complexities of managing daily care, along with the emotional demands of supporting a child's developmental and therapeutic needs, can lead to heightened levels of stress, anxiety and feelings of isolation among caregivers [1]. As the importance of caregiver well-being becomes increasingly recognised, there is a growing interest in identifying effective support strategies that can mitigate these challenges and enhance the overall quality of life for both caregivers and their children [2]. In recent years, the therapeutic benefits of animal companionship, particularly through pet dog ownership, have garnered significant attention across various populations. The bond between humans and dogs has been shown to have a positive impact on mental health, reducing stress, anxiety and depression while promoting social interaction, physical activity and emotional support [3]. For caregivers of children with intellectual disabilities, pet dogs may offer an additional layer of support, providing companionship not just to the child but also to the caregiver, potentially alleviating some of the emotional burdens associated with caregiving [4]. Despite the anecdotal evidence and emerging research supporting the benefits of pet ownership in caregiving contexts, there remains a need for comprehensive studies that explore the specific impact of pet dog ownership on the well-being of caregivers of children with intellectual disabilities [5]. Therefore, it is of interest to fill this gap by investigating the relationship between pet dog ownership and the emotional well-being, stress levels and anxiety of caregivers in this unique caregiving situation.

## Material and Methods:

The psychosocial impact of various events and things on humans (in this study, care caregivers of mentally challenged children) can be checked by multiple methods of scales, parameter tests, etc. In this study, we investigated the psychological and social impacts of pet ownership on caregivers, like fathers, mothers or any other in children with mental disorders. This Study was done in the Paediatrics Department of the District Hospital in Amritsar, Punjab, India and was carried out with the permission of the Ethics Committee and with the agreement of the children's legal guardians with the Institutional Review Board (IRB) number REF 12/03/2018. A total of 112 children were included in the study and were counselled. The research was carried out in compliance with the Declaration of Helsinki. 20 patients were lost to follow up and the pet ownership materialised in 52 patients. The study was divided into 2 groups. The compliant group (who owned a dog) included 52 children and the non-compliant group (who did not own a dog) was formed of 40 children. Hamilton Anxiety Scale (HAM-A) was applied to all the caregivers before pet dog ownership (PRE) and after 3-6 months with a pet dog (POST) [5]. The pre- and post-scores of all the subjects were recorded and subjected to statistical analysis. One of the first rating measures for anxiety symptoms, the HAM-A is still frequently used today in clinical and research settings. When it comes to assessing both mental and physical stress, the scale includes 14 things that each has their own symptoms, each of which may be described by the physical complaints related to anxiety. It has been predetermined that the results of the evaluation can be interpreted as follows: there is a total score range of 0-56 and a score of 17 or less indicates mild anxiety severity; a score from 18 to 24 indicates mild to moderate anxiety severity; and lastly, a score of 25 to 30 indicates a moderate to severe anxiety severity. The data were analysed using standard statistical formulas. Descriptive statistics such as mean and standard deviation were calculated for continuous variables. To assess the effect of pet dog ownership on anxiety levels, pre- and post-intervention HAM-A scores were compared within each group using the paired t-test. To compare the mean change in anxiety scores between the compliant and non-compliant groups, the independent sample t-test was used.

## Results:

In the compliant group of caregiver1, the mean anxiety score before pet ownership was 26.5±6.99, which significantly reduced to 19.53±4.64 after 3-6 months of pet ownership (p< 0.00001). The mean change in anxiety score between the two time intervals was 6.96±4.18, indicating a statistically significant reduction in anxiety. In the non-compliant group, the mean score before pet ownership was 25.5±6.69 and after 3-6 months was 24.72±6.53. Although there was a small reduction, it was less marked, with a mean change in score of 0.78±1.86, which was statistically significant (p=0.010462) but of lesser magnitude as compared to the compliant group. Between-group comparison showed a statistically significant difference in the reduction of anxiety scores, with the compliant group showing a significantly greater improvement (p<0.00001) Table 1. The results in Table 2 show that in the compliant group of caregiver 2, the mean anxiety score before pet ownership was 27.28±4.62, which was significantly reduced to 20±2.85 after 3-6 months of pet ownership (p<0.00001). The mean change in anxiety score was 7.28±3.78, indicating a significant reduction in anxiety. In contrast, the non-compliant group showed a mean anxiety score of 25.48±5.71 before pet ownership and 25.69±5.76 after 3-6 months with no statistically significant change (p=0.424689).The mean change in score for the non-compliant group was -0.21±1.63, which was not statistically significant. Comparison between the groups showed a highly significant difference in anxiety score reduction, with the compliant group demonstrating greater improvement (p<0.00001) Table 2.

## Discussion:

The study demonstrates a significant relationship between pet ownership and improved mental health outcomes for caregivers of children with autism spectrum disorder (ASD) and other intellectual disabilities. Notably, research has shown that pet ownership can lead to reduced stress levels, improved emotional well-being and enhanced social support for caregivers. Allen *et al.* interpreted in terms of the degree to which friends and pets are perceived as evaluative during stressful task performance [6]. Friedman & Thomas established that pet ownership and social support are significant predictors of survival among patients with coronary artery disease [7]. One key finding is that the presence of pets can serve as a buffer against stress and anxiety, providing emotional support and companionship to caregivers. Wilson confirmed that baseline differences in trait anxiety scores indicate a potentially greater benefit for pet owners than nonowners [8]. This is particularly important for parents of children with ASD, who often experience high levels of parenting stress [9]. A study describes the Stress levels of 38 primary carers acquiring a dog and 24 controls not acquiring a dog using the Parenting Stress Index. Analysis revealed significant improvements in the intervention compared to the control group for Total Stress, Parental Distress and Difficult Child. A significant number of parents in the intervention group moved from clinically high to normal levels of Parental Distress. Studies have indicated that pet ownership can mitigate some of this stress, contributing to better overall mental health for caregivers, Burgoyne *et al.* (2014) [10]. Furthermore, the therapeutic potential of pets extends beyond individual benefits. Pets can facilitate social interaction and provide a sense of purpose and meaning for caregivers, which can be especially valuable for those managing the challenges of caring for a child with intellectual disability [11]. The bond between humans and animals has been shown to have positive effects on mental health, including reduced symptoms of depression and anxiety by Odendaal & Meintjes [12].

## Conclusion:

The evidence suggests that pet ownership can have a profoundly positive impact on the well-being of caregivers of children with intellectual disabilities. By recognising and leveraging the therapeutic benefits of pets, we can enhance support systems for these caregivers and improve their overall quality of life.

## Figures and Tables

**Table 1:** Caregiver 1 group Analysis based on HAM-A score based on Hamilton Anxiety Scale, where*p<0.05 denotes significant results and **p<0.001 corresponds to highly significant results.

	**Compliant group**	**Non-compliant group**	**p value**
Before pet ownership	26.5±6.99	25.5±6.69	p=0.48831
After 3-6 months of pet ownership	19.53±4.64	24.72±6.53	p=0.000065
Change in score between two time intervals	6.96±4.18	0.78±1.86	p=<0.00001
p value	p=<0.00001	p=0.01462	

**Table 2:** Caregiver 2 group Analysis based on HAM-A score based on HAM-A: Hamilton Anxiety Scale, where *p<0.05 denotes significant results and **p<0.001 corresponds to highly significant results

	**Compliant group**	**Non-compliant group**	**p value**
Before pet ownership	27.28±4.62	25.48±5.71	p=0.11730
After 3-6 months of pet ownership	20.0±2.85	25.69±5.76	P<0.00001
Change in score between two time intervals	7.28±3.78	-0.21±1.63	p<0.00001
p value	p<0.00001	p=0.424689	
